# Spatio-temporal secondary instabilities near the Turing-Hopf bifurcation

**DOI:** 10.1038/s41598-019-47584-9

**Published:** 2019-08-02

**Authors:** Aldo Ledesma-Durán, José L. Aragón

**Affiliations:** Centro de Física Aplicada y Tecnología Avanzada, Universidad Nacional Autónoma de México, Boulevard Juriquilla 3001, 76230 Querétaro, Mexico

**Keywords:** Fluid dynamics, Nonlinear phenomena, Applied mathematics, Applied physics

## Abstract

In this work, we provide a framework to understand and quantify the spatiotemporal structures near the codimension-two Turing-Hopf point, resulting from secondary instabilities of Mixed Mode solutions of the Turing-Hopf amplitude equations. These instabilities are responsible for solutions such as (1) patterns which change their effective wavenumber while they oscillate as well as (2) phase instability combined with a spatial pattern. The quantification of these instabilities is based on the solution of the fourth order polynomial for the dispersion relation, which is solved using perturbation techniques. With the proposed methodology, we were able to identify and numerically corroborate that these two kinds of solutions are generalizations of the well known Eckhaus and Benjamin-Feir-Newell instabilities, respectively. Numerical simulations of the coupled system of real and complex Ginzburg-Landau equations are presented in space-time maps, showing quantitative and qualitative agreement with the predicted stability of the solutions. The relation with spatiotemporal intermittency and chaos is also illustrated.

## Introduction

Oscillatory patterns are recurrently observed in a variety of physical and chemical systems. In a reaction-diffusion (RD) system they usually occur when parameter values are close to the codimension-2 Turing-Hopf point (CTHP), *i*.*e*., the point in the parameter space where both bifurcations coincide. One way of studying the solutions in this regime is by means of the amplitude formalism, where an approximation is obtained by decomposing the solution in powers of a perturbation parameter that measures the distance to the CTHP and separating fast and slow spatiotemporal scales^1^. The result is a system of coupled real and complex equations of the Ginzburg-Landau (GL) type, where the real equation accounts for the spatial modulations of the steady spatial pattern (Turing mode), and the complex equation measures the modulations of the limit cycle solution (Hopf mode). The strength of the coupling increases as the system is near to CTHP and decreases as the parameters of the system approach to either the Turing or the Hopf bifurcation; in this last case, the system can be studied with either the real or the complex GL equations, respectively^2^. The importance of the amplitude formalism lies in its universal character to represent many dynamical systems near the CTHP and its capability for obtaining a Mixed Mode (MM) solution, combining the expected spatial and temporal periodicity. This MM solution was first experimentally verified in chemical systems^3^, and at the same time an important analytic understanding of its existence and stability in small domains was developed^4–6^. With this, the Turing-Hopf amplitude equations were proposed as approximate solutions of a variety of dynamical systems^7–9^.

Despite the importance of oscillatory patterns, only a few works use the amplitude formalism mainly because the study of the stability of the Mixed mode solution has been restricted to the homogeneous Turing and Hopf states, *i*.*e*. solutions where both modes have constant amplitudes, giving place to spatial pattern of fixed wave number oscillating in phase along the domain^10^. However, as occurs when real and complex GL equations are considered separately, it is expected that as the size of the domain increases, spatial modulations appear in the solution, producing changes in the effective wavenumber of the the Turing amplitude (Eckahus intability) or result in phase instability for the Hopf amplitude (Benjamin-Feir-Newel instability)^11^. It was always assumed that both instabilities should also occur for a codimension-two bifurcation^5^, although it was only recent that both instabilites were numerically identified in a RD system^12^.

The main goal of this work is to use the Turing-Hopf amplitude equations to obtain the conditions for which both Eckhaus and Benjamin-Feir-Newel (BFN) instabilities occur in a system near the CTHP. To achieve this, the Mixed mode solution is perturbed to obtain the dispersion relation for the eigenvalue that measures the growing of sideband modulations, using the standard procedure^13,14^. However, as it will be explained below, in contrast to the standard Eckhaus or BFN instabilities^11^, in this case the dispersion relation is a fourth order polynomial whose solutions are not only hard to obtain but it is even also difficult to grasp their physical significance.

Therefore, in this work, we use the perturbative method for solving algebraic equations^15,16^ for finding the roots of the dispersion relation. We will prove that two of these roots are related to generalizations of the Eckhaus and BFN instabilities for the Mixed Mode solution. These theoretical results are compared with the numerical solutions of the Turing-Hopf amplitude equations showing an excellent quantitative agreement. Besides, we will present a novel form of presenting the stability of the Mixed mode solutions in what we name *stability diagrams*, and explain how to use them for extract quantitative and qualitative conclusions of the possible solutions that the system can have as the size of the domain increases. These results allow us to extend our understanding of the solution of dynamical systems near the CTHP to more realistic physical situations where larger domains and spatial perturbations give place to secondary instabilities, intermittency and chaos.

The organization of the work is as follows. In Section Antecedents the basic features of the Turing-Hopf amplitude equations are presented, as well as the known facts about the secondary instabilities of the uncoupled real and complex GL equations. In Section Methodology we pose the problem of secondary instabilities in the coupled system of GL equations and solve the dispersion relation perturbatively. In section Numerical Results we numerically verify the stability of the solutions predicted by the stability diagrams. Besides this, the numerical solutions of the GL equations are used to make qualitative predictions on different dynamical systems. Finally, in Section Conclusions, the validity and general scope of our work is discussed.

## Antecedents

In the vicinity of the CTHP, oscillating patterns, steady spatial patterns and homogeneous oscillations can be found^5^. They are the result of a *primary instability*, that is, the first transition occurring in a dynamical system as the result of a bifurcation^13^; the Turing-Hopf bifurcation in this case. These three solutions can be described generally by assuming a *Mixed Mode* solution ${\bf{u}}(x,t)$, which combines the influence of both a spatial stationary pattern with wavenumber *k*_*c*_ and an homogeneous oscillation with angular frequency ${\omega }_{c}$:

1 $${\bf{u}}\approx T(x,t){e}^{i{k}_{c}x}{{\bf{u}}}_{T}+H(x,t){e}^{i{\omega }_{c}t}{{\bf{u}}}_{H}+{\rm{c}}.{\rm{c}}.,$$

where *H* and *T* are the *amplitudes* of the Hopf and Turing modes, respectively, measuring the weight of each mode in the solution, and c.c. denotes the complex conjugate. *k*_*c*_ and ${\omega }_{c}$ can be obtained from the analysis of the linearized system, whereas the spatiotemporal evolution of the amplitudes can be obtained by the method of multiscales, as a perturbation series in powers of the distance of two parameters of the system to the CTHP. At first order of approximation, amplitudes *T* and *H* obey the coupled system of GL equations

2a $$\frac{\partial T}{\partial t}={\mu }_{t}\,T-{a}_{t}|T{|}^{2}T+{b}_{t}\frac{{\partial }^{2}T}{\partial {x}^{2}}-{c}_{t}|H{|}^{2}T,$$

2b $$\frac{\partial H}{\partial t}={\mu }_{h}\,H-{a}_{h}|H{|}^{2}H+{b}_{h}\frac{{\partial }^{2}H}{\partial {x}^{2}}-{c}_{h}|T{|}^{2}H,$$

where *a*_*t*_, *b*_*t*_, *c*_*t*_ are the real coefficients of the Turing mode equation, and *a*_*h*_ = *α*_*r*_ + *iα*_*i*_, *b*_*h*_ = *β*_*r*_ + *iβ*_*i*_ and *c*_*h*_ = *γ*_*r*_ + *iγ*_*i*_ are the complex coefficients of the equation for the wave mode^1^. *μ*_*t*_ and *μ*_*h*_ are proportional to the distance of two parameters to the CTHP. The approximate solution derived from the solution of (2) in (1), describes the spatiotemporal solutions of a great variety of dynamical systems near the Turing-Hopf bifurcation. Given this generality of the amplitude equations, the peculiarity of each dynamical system will be reflected in the coefficients of the approximated equations in (2), which depend on the parameters of the original system.

The system (2) has three known families of solutions: (1) pure Turing structures, (2) plane waves, and (3) MM solutions, where both the spatial pattern and wave propagation occur^3^. The general form of the solution of (2) is

3 $$T(x,t)=Z{e}^{iRx},\,H(x,t)=A{e}^{iQx-i{\rm{\Omega }}t}.$$

where *Z*, *A* and ${\rm{\Omega }}$ depend on the wavenumbers *R* and *Q*^3^ as:

4a $$Z=\sqrt{\frac{({\mu }_{t}-{b}_{t}{R}^{2})-\tau ({\mu }_{h}-{\beta }_{r}{Q}^{2})}{(1-\tau \sigma ){a}_{t}}},$$

4b $$A=\sqrt{\frac{({\mu }_{h}-{\beta }_{r}{Q}^{2})-\sigma ({\mu }_{t}-{b}_{t}{R}^{2})}{(1-\tau \sigma ){\alpha }_{r}}},$$

4c $${\rm{\Omega }}={\alpha }_{i}{A}^{2}+{\beta }_{i}{Q}^{2}+{\gamma }_{i}{Z}^{2},$$

where $\tau \equiv {c}_{t}/{\alpha }_{r}$ and $\sigma \equiv {\gamma }_{r}/{a}_{t}$. When *Z* ≠ 0 and *A* ≠ 0, (8) and (4) define the MM solution^3^. The pure Turing mode is recovered in the limit $\tau \to 0$ (${\alpha }_{r}\to \infty$) where

5 $${A}_{Turing}^{2}=0\,{\rm{and}}\,{Z}_{Turing}^{2}=\frac{({\mu }_{t}-{b}_{t}{R}^{2})}{{a}_{t}},$$

and the pure wave solution is recovered when $\sigma \to 0$ (${a}_{t}\to \infty$), where the amplitudes are^1^

6 $${A}_{Hopf}^{2}=\frac{({\mu }_{h}-{\beta }_{r}{Q}^{2})}{{\alpha }_{r}}\,{\rm{and}}\,{Z}_{Hopf}^{2}=0.$$

These limiting cases ($\tau \to 0$ and $\sigma \to 0$) will be important below to identify the weak coupling limit.

The conditions for the *existence* of a MM solution require positive radicands in (4a) and (4b), which at the same time define the *marginal* conditions for *R* and *Q*. In particular, for the *homogeneous state*, *Q* = *R* = 0, from (4) we get that the existence conditions of the MM solution are

7 $$\frac{1-\tau {\delta }^{-1}}{1-\tau \sigma }(\frac{{\mu }_{t}}{{a}_{t}}) > 0\,{\rm{and}}\,\frac{1-\delta \sigma }{1-\tau \sigma }(\frac{{\mu }_{h}}{{\alpha }_{r}}) > 0,$$

respectively. Since *μ*_*t*_ and *μ*_*h*_ measure the distance to their respective bifurcation, then $\delta \equiv {\mu }_{t}/{\mu }_{h}$ is a measure of the distance to both bifurcations since the cases |*δ*| ≈ 0 and $|\delta |\gg 1$ reflect more proximity of the system to the Turing or to the Hopf bifurcation, respectively. In what follows it will be assumed that existence conditions of the MM solution in (7), are fulfilled.

Equation (3) are exact solutions of (2) at some infinite set of the parameters, provided that the existence conditions stated before are fulfilled. However, near the CTHP, the occurrence of one or other solution among the three available families will depend upon their respective stability, which in turn it is expected to depend upon the proximity of the system to either one or another bifurcation. There are some few examples of RD systems where the stability of the solutions has been numerically studied^5,8,9,12^. However, up to now, the formal analysis is known only for the homogeneous state. As it is clear from substituting $R=Q=0$ in Eq. (3), this corresponds to a spatial pattern of constant wavenumber oscillating in time with constant phase^6^. This is the expected solution on very small domains or in systems where no spatial perturbations occur^10^.

However, for most of the relevant systems, it is expected that inhomogeneous perturbations can cause also inhomogeneous states of the solution, *i*.*e*. states of the MM solution where *R* or *Q* are different from zero^12^. The effect of these inhomogeneous perturbations can be understood by substituting (3) in (1):

8 $${\bf{u}}\approx Z{e}^{i({k}_{c}+R)x}{{\bf{u}}}_{T}+A{e}^{iQx+i({\omega }_{c}-{\rm{\Omega }})t}{{\bf{u}}}_{H}+{\rm{c}}.\,{\rm{c}}.\,,$$

where it is clear that *R* ≠ 0 accounts for a change in the effective wavenumber of the final spatial pattern respect to *k*_*c*_, and *Q* ≠ 0 produces a phase destabilization of the temporal solution and results in a wave behavior of the solution or even in phase chaos. We can consider these two types of solutions as *secondary instabilities*, that is, the result of a second transition to a new regime where the previous state (in our case, a oscillatory pattern of frequency ${\omega }_{c}$ and wavenumber *k*_*c*_), is in itself the result of a bifurcation (in our case, the Turing-Hopf bifurcation).

As it was expected^5^ and numerically corroborated^12^, the change of effective wavenumber and the phase unstabilization of the Mixed Mode, explained in (8), can be understood as generalizations of the Eckhaus and BFN instabilities previously described for the real and complex uncoupled GL equations separately^11^. Since our results on the analysis of (2) must recover these two well-known instabilities in the limit of zero coupling between Turing and Hopf modes, let us briefly consider the secondary instabilities of the real and complex GL equations separately.

If the system is near a Turing bifurcation but away from the Hopf bifurcation, the system obeys (2a) with *c*_*t*_ = 0, *i*.*e*., the real GL equation. The solution of the system is a pattern with wavenumber *k*_*c*_ + *R*, where the values of *R* for stability are determined by *Eckhaus stability criterion*:

9 $${\lambda }_{Eck}\cong -\,{b}_{t}[1-\frac{2{b}_{t}{R}^{2}}{{\mu }_{t}-{b}_{t}{R}^{2}}]\,{k}^{2} < 0,$$

where *λ*_*Eck*_ measures the temporal growth of sideband perturbations with wavenumber *k*^11^. In the supercritical regime *b*_*t*_, *μ*_*t*_ > 0, thus solutions with wavenumbers *R* are stable if *R* < *R*_*Eck*_ with ${R}_{Eck}^{2}\equiv {\mu }_{t}/(3{b}_{t})$^17^. Perturbations with larger wavenumbers can be destabilized if the domain size is large enough, since the periodic spatial solution admits only an integer number of wavelengths.

When the system is near a Hopf bifurcation, the system obeys (2b) with *c*_*h*_ = 0, *i*.*e*., the complex GL equation. In this case, the solution of the system is a plane wave with phase $\varphi =Qx-{\rm{\Omega }}t$. The temporal growth *λ*_*BFN*_ of sideband perturbations of wavenumber *k* is determined by

10 $${\lambda }_{BFN}\cong -\,i{v}_{g}k+{D}_{\parallel }{k}^{2}+{\mathscr{O}}({k}^{3}),$$

where the first term of the right hand side is proportional to the group velocity of the wave, ${v}_{g}=2Q{\beta }_{r}(\beta -\alpha )$, and the second term provides the *Benjamin-Feir-Newell criterion of stability* given by

11 $${D}_{\parallel }\equiv -\,{\beta }_{r}[(1+\alpha \beta )-\frac{2{Q}^{2}{\beta }_{r}(1+{\alpha }^{2})}{({\mu }_{h}-{\beta }_{r}{Q}^{2})}] < 0,$$

where $\alpha \equiv {\alpha }_{i}/{\alpha }_{r}$ and $\beta \equiv {\beta }_{i}/{\beta }_{r}$. In the supercritical regime, the stability of the wave requires *β*_*r*_ > 0 and (1 + *αβ*) > 0, and the critical wavenumber is ${Q}_{BFN}^{2}\equiv [(1+\alpha \beta )/(3+2{\alpha }^{2}+\alpha \beta )]\,({\mu }_{h}/{\beta }_{r})$. Phase instability can arise when *Q* > *Q*_*BFN*_^2^.

Recently, we have numerically identified these solutions in a RD system close to the CTHP, and shown that, in the Eckhaus instability, a change in the wavenumber of the spatial pattern occurs while the entire solution oscillates in phase and, in the case of the BFN phase instability, a spatial pattern with fixed positions of crests and troughs is combined with the propagation of a wavefront that change their height^12^. We also claimed that it is necessary to take into account inhomogeneous perturbations since the current analysis, which considers only homogeneous states, overestimates the regions of stability of pure Hopf and Turing solutions. This means that oscillatory spatial pattern can reach larger regions of the parameter space as the size of the domain is increased.

Since patterns arising close to the CTHP point have been frequently reported in experimental^18–20^ and numerical^21–23^ investigations and some of them could be the result of such secondary instabilities of the MM solution, in this work we provide a methodology to understand and quantify the stability of inhomogeneous states of the MM solution of (1). In the next section we describe a methodology to study these structures in a very general framework and to establish their stability conditions in terms of the system parameters.

## Methodology

The purpose of this work is to quantify the secondary instabilities of the Mixed mode solution. Thus, the growth of inhomogeneous perturbations *λ* will be measured by following the standard procedure described in ref.^11^, where secondary instabilities of the real and complex GL equations are quantified separately. Our purpose is to obtain and solve the dispersion relation in order to identify how each secondary instability of the Turing and Hopf modes, given by (9) and (11) separately, is modified by the presence of the other mode. Since this analysis requires to find the solutions of a fourth order polynomial for *λ*(*k*), we will use a standard perturbative method for solving algebraic equations, where a parameter (in our case, the wavenumber of the sideband perturbation *k*) is small^15,16^. As we will see, with this approach a stability diagram where the kind of stability of the MM solutions with given *R* and *Q* in (3) can be theoretically predicted.

To find the secondary instabilities of the MM solution, we consider the sideband modulations of the harmonics^11^. The solutions (3) are perturbed as

12a $$H(x,t)=(A+p{e}^{ikx+\lambda t}+q{e}^{-ikx+\bar{\lambda }t}){e}^{iQx-i{\rm{\Omega }}t},$$

12b $$T(x,t)=(Z+r{e}^{ikx+\lambda t}+s{e}^{-ikx+\bar{\lambda }t}){e}^{iRx},$$

where *p*, *q*, *r* and *s* are complex numbers and the overline denotes the complex conjugate. By substituting (12) in (2), and neglecting non linear terms, we obtain the eigenvalue problem (**M** − *λ***I**)**x** = 0, where ${\bf{x}}={(p,\bar{q},r,\bar{s})}^{T}$ is the vector of perturbations, **I** is the 4 × 4 identity matrix and **M** is the matrix of coefficients with diagonal entries:

$$\begin{array}{rcl}{M}_{11} & = & -\,2{a}_{h}{A}^{2}-{b}_{h}{(k+Q)}^{2}+i{\rm{\Omega }}-{c}_{h}{Z}^{2}+{\mu }_{h},\\ {M}_{22} & = & -\,2{\bar{a}}_{h}{A}^{2}-{\bar{b}}_{h}{(k-Q)}^{2}-i{\rm{\Omega }}-{\bar{c}}_{h}{Z}^{2}+{\mu }_{h},\\ {M}_{33} & = & -\,2{a}_{t}{Z}^{2}-{c}_{t}{A}^{2}-{b}_{t}{(k+R)}^{2}+{\mu }_{t},\\ {M}_{44} & = & -\,2{a}_{t}{Z}^{2}-{c}_{t}{A}^{2}-{b}_{t}{(k-R)}^{2}+{\mu }_{t}.\end{array}$$

The remaining entries are $${\bar{M}}_{21}={M}_{12}=-\,{a}_{h}{A}^{2}$$, $${M}_{14}={M}_{13}=-\,{c}_{h}AZ$$, $${M}_{23}={M}_{24}={\bar{M}}_{13}$$, $${M}_{31}={M}_{32}={M}_{41}=$$$${M}_{42}=-\,{c}_{t}AZ$$ and $${M}_{34}={M}_{43}=-\,{a}_{t}{Z}^{2}$$. The *dispersion relation* of the eigenvalue problem can be written as

13 $${\lambda }^{4}+{f}_{3}(k){\lambda }^{3}+{f}_{2}(k){\lambda }^{2}+{f}_{1}(k)\lambda +{f}_{0}(k)=0,$$

where *f*_*i*_(*k*) are polynomials as functions of the perturbation parameter *k*. In general, (13) has four possible roots, which represent the different behaviors of the solutions in (12). We will show that two roots are zero when *k* = 0 and, therefore, constitute bifurcations. It turns out that these two solutions correspond to generalizations of *λ*_*Eck*_ and *λ*_*BFN*_ given in (9) and (11), respectively. The stability criteria of the MM solution to homogeneous conditions will be obtained from the remaining two solutions.

The roots of (13) are difficult to obtain and their exact values are complicated expressions from which no useful conclusions can be derived. In view of this, the idea of the perturbation method is to express the eigenvalue *λ* and the functions *f*_*i*_ in (13) as a power series of the perturbation parameter *k* (assumed small) and then, solve algebraic equations recurrently. This will allow us to compute the solutions of *λ* up to second order, and compare with the Eckhaus and BFN criteria calculated in (9) and (11). Therefore, we expand *λ* and *f*_*i*_ as

14 $$\lambda ={\lambda }_{0}+{\lambda }_{1}k+{\lambda }_{2}{k}^{2}+\ldots ,\,{\rm{and}}\,{f}_{i}(k)={f}_{i0}+{f}_{i1}k+{f}_{i2}{k}^{2}+\ldots $$

By substituting in (13), the result can be arranged in powers of *k* as ${P}_{0}({\lambda }_{i})+{P}_{1}({\lambda }_{i})k+{P}_{2}({\lambda }_{i}){k}^{2}+\cdots =0$, where each coefficient *P*_*j*_ is a function of *λ*_0_, *λ*_1_, …, *λ*_*j*_. Thus, if ${P}_{j}(\{{\lambda }_{i}\})=0$ is equated to zero, the system can be solved recurrently. The values of the coefficients *f*_*ij*_ are given in the Supplementary Material.

Since in our case ${f}_{00}={f}_{10}=0$, the zero order solution is obtained from

15 $${P}_{0}({\lambda }_{0})={\lambda }_{0}^{2}({f}_{20}+{f}_{30}{\lambda }_{0}+{\lambda }_{0}^{2})=0.$$

From this equation it is clear that *λ*_0_ = 0 is a double root and the two corresponding eigenvalues define two instabilities when high order terms are included. Besides, since the remaining coefficients in ${f}_{20}+{f}_{30}{\lambda }_{0}+{\lambda }_{0}^{2}$ are real, the other two solutions for *λ*_0_ are negative when *f*_20_ and *f*_30_ are positive. This guarantees that the two solutions with *λ*_0_ = 0 are the greatest eigenvalues at order *k* = 0. It can be proved that *f*_20_, *f*_30_ > 0 when

16 $$4{A}^{2}{Z}^{2}(1-\tau \sigma ){\alpha }_{r}{a}_{t} > 0,\,{\rm{and}}\,2[{\alpha }_{r}{A}^{2}+{a}_{t}{Z}^{2}] > 0.$$

In this case, the two stable eigenvalues at order *k* = 0 are ${\lambda }_{0}=(1/2)\,(\,-\,{f}_{30}\pm \sqrt{{f}_{30}^{2}-4{f}_{20}})$, which do not correspond to bifurcation eigenvalues, so they will be ignored. However, as the conditions in (16) are determined at zero order in *k* in (12), they determine the stability to homogeneous perturbations of the MM solution. Actually, (7) and (16) were deduced in ref.^10^ as *existence and stability conditions of the homogeneous MM solution*.

For higher orders in *k*, we see that nothing new is obtained at first order, since $${P}_{1}({\lambda }_{0},{\lambda }_{1})$$ = $${\lambda }_{0}$$($${f}_{11}+{f}_{21}{\lambda }_{0}+$$$${f}_{31}{\lambda }_{0}^{2}+{f}_{20}{\lambda }_{1}+{f}_{30}{\lambda }_{0}{\lambda }_{1}+4{\lambda }_{0}^{2}{\lambda }_{1}$$) is proportional to *λ*_0_ and we are interested in the bifurcation solutions with *λ*_0_ = 0.

At second order, we use the fact that ${f}_{02}={f}_{10}=0$ to obtain ${P}_{2}({\lambda }_{0}=0,{\lambda }_{1})={\lambda }_{1}({f}_{11}+{f}_{20}{\lambda }_{1})$, which does not depend on *λ*_2_. Therefore, there are two solutions for *λ*_1_, namely *λ*_1_ = 0 and ${\lambda }_{1}=-\,{f}_{11}/(2{f}_{20})$. This means that

17 $${\lambda }_{1}^{Eck}=0\,{\rm{or}}\,{\lambda }_{1}^{BFN}=\frac{2iQ{\beta }_{r}(\alpha -\beta )\,(1-\tau \sigma \chi )}{1-\tau \sigma },$$

where

18 $$\chi \equiv \frac{\gamma -\beta }{\alpha -\beta },$$

and $\gamma \equiv {\gamma }_{i}/{\gamma }_{r}$. The labels *Eck* and *BFN* are included since these eigenvalues will be related to such instabilities, when higher orders in *k* are included. At order *k*, the traditional Eckhaus eigenvalue in (9) is 0 as in (17); in the same way, the group velocity of the wave solution in (10), is recovered from (17) in the limit $\sigma \to 0$.

Since *f*_10_ = 0, at order *k*^3^ the polynomial *P*_3_ depends only on *λ*_0_ = 0, *λ*_1_ and *λ*_2_. Therefore, it should be calculated for each value of *λ*_1_ obtained in the previous paragraph.

For the Eckhaus related eigenvalue, ${P}_{3}({\lambda }_{0}=0,{\lambda }_{1}={\lambda }_{1}^{Eck},{\lambda }_{2})={f}_{03}+{f}_{11}{\lambda }_{2}$. From *P*_3_ = 0 we obtain

19 $${\lambda }_{2}^{Eck}=-\,b[1-\frac{2{b}_{t}{R}^{2}}{{Z}^{2}(1-\tau \sigma \chi ){a}_{t}}].$$

Since ${\lambda }_{1}^{Eck}=0$, one eigenvalue at order *k*^2^ is $\lambda ={\lambda }_{2}^{Eck}{k}^{2}$. To describe this instability, consider the low interaction limit of the Hopf and Turing modes where $\tau \to 0$ and ${Z}^{2}\to {Z}_{Turing}^{2}$ in (5). In this case, we recover Eckhaus stability criterion in (9) for the real GL equation^17^. Notice however, that the Eckhaus criterion depends now on *Q*, the wavenumber of the wave solution, through *Z*. Our analysis predicts that this instability depends also on *χ* in (18), which is related to the complex coefficients of the Hopf mode, *α*, *β* and *γ*.

For the BFN related eigenvalue, ${P}_{3}({\lambda }_{0}=0,{\lambda }_{1}={\lambda }_{1}^{BFN},{\lambda }_{2})$ = ${f}_{03}-({f}_{11}({f}_{12}{f}_{20}^{2}-{f}_{11}{f}_{20}{f}_{21}+{f}_{11}^{2}{f}_{30}+{f}_{20}^{3}{\lambda }_{2}))$/${f}_{20}^{3}$. The complete substitution is given at the Supplementary Material, but at first order in *σ*, it turns out to be

20 $${\lambda }_{2}^{BFN}\cong -\,{\beta }_{r}[(1+\alpha \beta )-\frac{2(1+{\alpha }^{2})\,({\mu }_{h}-{\alpha }_{r}{A}^{2}-\sigma {a}_{t}{Z}^{2})}{{\alpha }_{r}{A}^{2}}].$$

Since ${\lambda }_{1}^{BFN}$ is a pure imaginary number, then ${\lambda }_{2}^{BFN}$ provides the first real part of the second eigenvalue $\lambda ={\lambda }_{1}^{BFN}k+{\lambda }_{2}^{BFN}{k}^{2}$. To understand this eigenvalue, consider the limit of null interaction with the Turing mode (*σ*, $Z\to 0$ and $A\to {A}_{Hopf}$). In this case, we recover the BFN criterion (10) of the complex GL equation^2^. However, in our case, due to the proximity with the CTHP, both modes interact and the stability of the spatial pattern and wave solutions depend on each other.

From (20) it is straightforward to deduced that the origin is a critical point where ${\lambda }_{2}^{BFN}(R=0,Q=0)$ is negative if

21 $${\beta }_{r}\frac{(1+\alpha \beta )-\tau \sigma (1+\beta \gamma )}{1-\tau \sigma } > 0$$

This criterion was deduced in ref.^4^ for the *phase stability of the homogeneous MM solution*.

In conclusion, we have proved that the *generalization of the Eckhaus instability near the CTHP* in (8) with *R* ≠ 0, represents a roll pattern solution, with an effective wavenumber moving from *k*_*c*_ to *k*_*c*_ + *R* when *R* and *Q* are inside the stability region given by the condition ${\lambda }_{2}^{Eck} < 0$ in (19). This change occurs while the entire solution oscillates in time or propagates slowly as a wave^24^. Clearly, ${\lambda }_{Eck}(R=0,Q=0)=-\,{b}_{t}{k}^{2}$, and therefore the destabilization of the system occurs when *b*_*t*_ ≤ 0 as in the traditional Eckhaus criterion (9).

We also proved that the second approximated eigenvalue solution of (13) has a real and a pure imaginary part. The imaginary part, given by ${\lambda }_{1}^{BFN}$ in (17), is related to the group velocity of the wave when *Q* ≠ 0 in (8). The unstable values of *R* and *Q* are defined by the condition ${\lambda }_{2}^{BFN} > 0$. This *generalization of the Benjamin-Feir-Newell instability* near the CTHP establishes the phase destabilization of the system into wave undulations or phase chaos combined with a spatial pattern. Therefore, this route to chaos has a semi-organized background due to the interaction with the Turing mode^25–28^.

The solutions near the CTHP are both Eckhaus and BFN stable when ${\lambda }_{2}^{Eck}$, ${\lambda }_{2}^{BFN} < 0$, respectively. These approximated criteria where calculated up to order *k*^2^ in (14). However, since *f*_10_ = 0, this process can be continued to higher orders in *k*. In particular, the fourth order approximation can be important to determine if the instabilities are of the long-wave type^2^.

Now, for the regular Eckhaus instability in the real GL equation, the marginal and stability conditions are usually represented in the (*μ*_*t*_, *R*) plane as two parabolas. With this plot, the number of possible stable and unstable wavenumbers *R*, as function of the distance to the bifurcation *μ*_*t*_, can be measured. In our case, however, the visualization of the results is complicated by the fact that for the MM solution, there are two distances to the bifurcation and two wavenumbers indexing the solution, *R* and *Q*, and therefore, such a plot should be four-dimensional.

**Figure 1:**
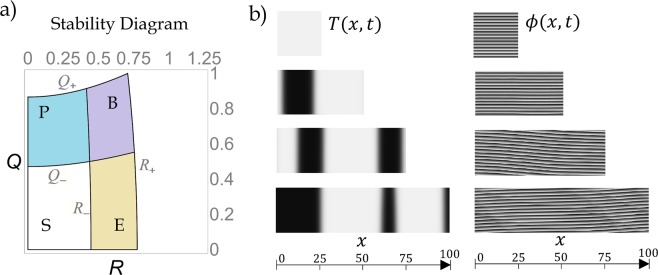
(**a**) Stability diagram of the solutions with wavenumbers *R* and *Q* in (8) predicted in this work and summarized in (4), (16), (19) and (20). The regions in the stability diagram are labeled as S (stable), E (Eckhaus unstable), P (Phase instability) and B (both Eckhaus and BFN unstable). Parameters of (2) are *μ*_*t*_ = 0.5, *a*_*t*_ = 2, *b*_*t*_ = 1, *c*_*t*_ = −0.1, *α*_*i*_ = 0.5, *β*_*i*_ = 0.1 and *γ*_*i*_ = 0.5. For this and subsequent figures we use *μ*_*h*_ = *α*_*r*_ = *β*_*r*_ = *γ*_*r*_ = 1 (**b**) Illustration of both instabilities. (**a**) Spacetime maps of *T* and $\varphi$ resulting from the numerical solution of (2) in four one-dimensional domains of different length *L*. Each row represents a different domain whereas the total time is *t*_*max*_ = 250. In this plot transient times were skipped and only the last interval of 80 units is shown (vertical).

However, for a given set of parameters (*i*.*e*. for given *μ*_*h*_ and *μ*_*t*_), a level curve of the existence conditions in (4) and of the Eckhaus and BFN stability conditions given in (19) and (20) can be plotted as function of *R* and *Q*. An example of this *stability diagram* is given in Fig. 1a. Each point in this plot represents a MM solution given in (3), in terms of the wavenumbers *R* and *Q*. Given the even symmetry of the stability and existence conditions on *R* and *Q*, only the first quadrant is plotted. The outer curves *R*_+_ and *Q*_+_ are determined by the existence conditions (4), whereas the inner curves *R*_−_ and *Q*_−_ depend on the stability conditions in (19) and (20).

These conditions determine four regions, where the MM solution exists: 1) The region of stability, where MM solution with initial conditions in (3) will stay the same even under small random perturbations; 2) The region of Eckhaus unstable solutions, where the MM solution will keep the phase with constant slope *Q*, but the wavenumber of the Turing mode will decay to a band of stable wavenumbers; 3) The region of BFN unstable solutions, where MM solution in (3) will keep the Turing wavenumber but the phase will lose the wavenumber *Q* of the initial condition and, finally, 4) The region where both instabilities occur and, therefore, the initial pattern with wavenumbers *R* and *Q* in (3) will change when random perturbations are applied. In the next section, we will provide quantitative and qualitative evidence of the validity of (19) and (20) and consequently, of the stability diagrams thus constructed.

## Numerical Results

Up to now, our understanding of the spatiotemporal solutions resulting from the eigenvalues in (19) and (20) has been given through the analogy with the well-known Eckhaus and BFN instabilities. In this Section, numerical evidence of our theoretical findings is provided. Therefore, the Turing-Hopf amplitude equations in (2) are solved in a one-dimensional domain, using the finite element software COMSOL Multiphysics*™*, with an adaptative mesh initialized with of 100 evenly spaced nodes, with periodic boundary conditions, and a time step Δ*t* = 0.025 used in all simulations.

An example of these solutions is in Fig. 1b, where we verify the presence of spatial modulations of the amplitude of the Turing mode *T*(*x*, *t*) which is a real number and the destabilization of the phase $\varphi (x,t)$ of the Hopf mode $H(x,t)=|H|{e}^{i\varphi }$ when the domain size is increased. In these spacetime maps, Eckhaus and BFN instabilities are easily identified as inhomogeneities in the spatial (horizontal) coordinate of *T*(*x*, *t*) and in the temporal (vertical) coordinate of $\varphi (x,t)$, respectively. In ref.^12^, we illustrate how these instabilities can be identified in spacetime maps and the phase space of a RD system.

Figure 1b shows that a mixed mode solution with *R* = *Q* = 0 is stable for small domains. Our analytic formalism predicts that for these parameter values there exists a critical size of the domain where the solutions do not change their wavenumber and oscillate in phase. We also corroborate that some modes of the solution lost Eckhaus stability when *L* ≈ 35, where the amplitude *T*(*x*, *t*) is not longer constant. This is displayed as black and white stripes in *T*(*x*, *t*) which correspond to spatial modulations of the Turing solution. Besides, if the system size is increased further to *L* ≈ 55, the Hopf solution lost BFN stability and the phase changes. This instability is displayed as undulations in the temporal solution of $\varphi (x,t)$, where the slope of the phase is no longer constant. In this case, the modulus of the Hopf mode |*H*| (*x*, *t*), not shown here, remains constant almost everywhere, except for small bumps or shocks near the sites where the Turing amplitude changes its sign. Since in these plots we try to illustrate the emergence of secondary instabilities as the size of the domain increases, we have used random initial conditions in a large domain, in such a way that several modes of the initial random perturbation can be unstabilized simultaneously. In this way, the behaviour of a dynamical system, not prepared with any particular choice of initial conditions but with a domain long enough to allow the presence of the Eckhaus and BFN instabilities, can be studied.

However, in order to validate the quantitative relationship between the numerical solutions of (2) and the stability diagram of Fig. 1a, the stability of the MM solutions, with wavenumbers *R* and *Q*, should be tested by initializing the system with (3) plus a small random perturbation in the space coordinate. If the solution for *T* at the simulation total time *t*_*max*_ remains with the same wavenumber *R*, then the solutions are Eckhaus stable. If the solution with wavenumbers *R* and *Q* is Eckhaus unstable, then the solution of *T* decays to a band of stable wavenumbers. The same can be said for the BFN stability if the solution for *H* remains with the same initial wavenumber *Q*.

**Figure 2:**
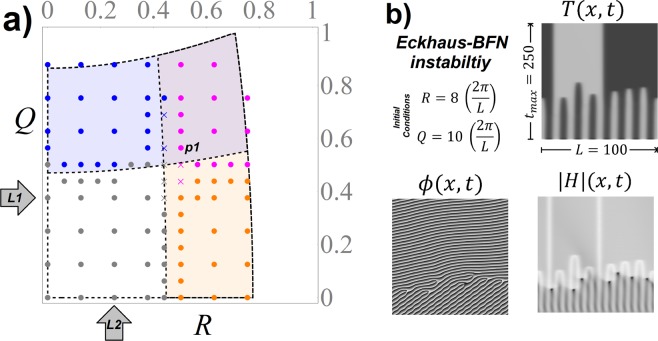
(**a**) Comparison of the theoretical stability diagram plotted in Fig. 1a, where the four types of solutions are presented by colored regions, with the numerical simulations of (2), with initial conditions given by (3) plus a spatial random perturbation. Each point represents a combination of initial wavenumbers (*R*, *Q*) and its color represents the stability of the numerical soluation: Stable (gray), Eckhaus unstable (orange), BFN unstable (blue) and Both instabilities are present (magenta). The crosses depict transition solutions, where the type of stability of the solution is uncertain. The space time maps of the Eckhaus-BFN unstable solution, represented by the point *p*1, is detailed in Fig. 2b, and the transition from Eckhaus and BFN stable to unstable patterns along the lines *L*1 and *L*2 is presented in Figs. 3a,b respectively. (**b**) Space time maps of the numerical solution of (2), where the initial conditions are in the Eckhaus-BFN unstable region.

**Figure 3:**
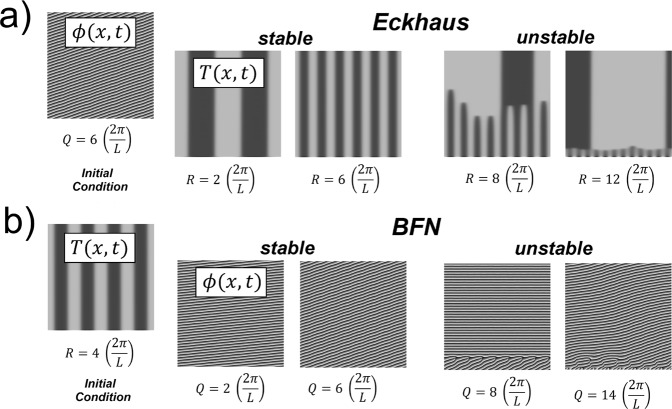
(**a**) Space-time maps of the numerical solutions of (2), where the system goes from the stable to the Echkaus unstable region along the line *L*1 in Fig. 2a. (**b**) The same for the transition to the BFN unstable region along *L*2. Notice that, in contrast with Fig. 2b, in this case the solution of the other mode (at the left) remains stable for all cases. As in Fig. 2, space and time run along the horizontal and vertical coordinates, respectively.

In Fig. 2a, the results of the stability for the numerical solutions of the system is shown for a particular set of parameters given at Fig. 1. Each point corresponds to a particular choice of initial conditions with wavenumbers (*R*, *Q*) in (3). In order to simplify the analysis, the size of the domain has been set to *L* = 100 and, therefore, multiples of wavenumbers 2*π*/*L* can be used to construct initial conditions of *R* and *Q*. In this way, changes in the wavenumber of the solutions for *T* and *H* can be easily identified by changes in the Fourier modes of the solution at *t*_*max*_ = 250. As it can be seen in Fig. 2a, the correspondence between the theoretical and numerical results is pretty good. The transition from stable to unstable regions is illustrated in Fig. 3, where the BFN instability is identifiable by the change in the slope of constant phase $\varphi (x,t)$, and the Eckhaus instability by the desapparition of uniform rolls and the apparition of strong plateaus in *T*(*x*, *t*). Both behaviors are present when the solution is in the Eckhaus-BFN unstable region as illustrated in Fig. 2b.

In this way, we have shown the agreement of the theoretical and numerical results of the secondary instabilities in Fig. 2a, for a given set of parameters where the distances to the Turing-Hopf bifurcation remain fixed. The full understanding of the validity of the instabilities in (19) and (20) have to be extended to different set of parameters varying such distances. This will allow to measure the validity of the amplitude formalism as function of the proximity to the CTHP. This study requires a detailed analysis of the Fourier modes of the Turing and Hopf solutions for different stability diagrams, which requires future numerical investigations.

In what follows, we focus on showing that the existence and stability conditions given in (4), (19) and (20), produce different arrangements of the stability diagrams. The idea is to explain how the features of these plots can be used to understand diverse qualitative properties of the solutions of the GL equations in (2), in terms of the domain size. In particular, we are interested in showing that the domain sizes where instabilities occur are intrinsically related to the stability diagram, since the critical domain size for the occurrence of any instability is inversely proportional to the unstable values of *R* or *Q*.

**Figure 4:**
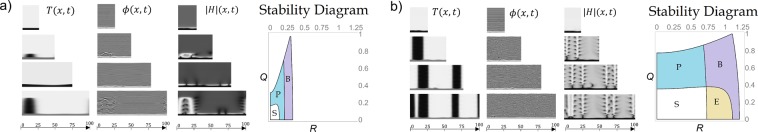
(**a**) Space time map solutions of (2) with parameter values: *μ*_*t*_ = 1, *a*_*t*_ = 1.111, *b*_*t*_ = 11.3, *c*_*t*_ = −0.2, *α*_*i*_ = 0.5, *β*_*i*_ = 0.1, *γ*_*i*_ = 1.3. (**b**) The same but for *μ*_*t*_ = 1, *a*_*t*_ = 2.5, *b*_*t*_ = 0.825, *c*_*t*_ = −0.2, *α*_*i*_ = 0.9, *β*_*i*_ = 0.1, *γ*_*i*_ = 2.5.

**Figure 5:**
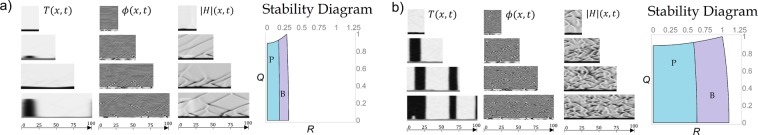
(**a**) Space time map solutions of (2) with parameter values *μ*_*t*_ = 1, *a*_*t*_ = 5, *b*_*t*_ = 12.5, *c*_*t*_ = −0.2, *α*_*i*_ = 1.1, *β*_*i*_ = −1, *γ*_*i*_ = 1.1. (**b**) The same but for *μ*_*t*_ = 1, *a*_*t*_ = 5, *b*_*t*_ = 1, *c*_*t*_ = −0.2, *α*_*i*_ = 1.5, *β*_*i*_ = −1.5, *γ*_*i*_ = 1.5.

**Figure 6:**
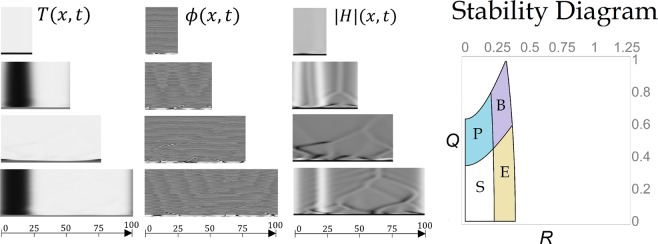
Space time map solutions of (2) with parameter values *μ*_*t*_ = 1, *a*_*t*_ = 1.666, *b*_*t*_ = 10, *c*_*t*_ = −0.5, *α*_*i*_ = 0.5, *β*_*i*_ = 0.1, *γ*_*i*_ = 0.5.

All subsequent figures (Figs 4–6) show the spacetime solutions maps of the real and complex GL equations (2), solved for the amplitude of the Turing mode, and for the phase and modulus of the Hopf mode. As in Fig. 1, each row corresponds to a different domain size, namely, *L* = 25, 50, 75 and 100 and the same random initial conditions are used for illustrative purposes. In all these figures, the transient times are displayed in the vertical direction up to a total time *t*_*max*_ = 100. In these plots, some spatio-temporal structures absent in the real and complex GL equations, separately, can be distinguished. The parameter values for each figure are given in the legend. The stability diagram of the solution with wavenumbers *R* and *Q* is also shown.

In Fig. 4 we contrast two examples where the critical size for the Eckhaus instability is very different. In the case of Fig. 4a, this instability cannot occur independently of the BFN instability and requires larger domain sizes. This is explained by the fact that the critical domain size for Eckhaus is inversely proportional to the unstable values of *R*. In contrast, the Eckhaus instability in Fig. 4b occurs for relatively small domains. Notice that in both cases, the coupling between the Turing and Hopf modes causes that the modulus |*H*(*x*, *t*)| exhibits interesting behaviors just where *T*(*x*, *t*) = 0, *i*.*e*. the vertical lines separating black and white zones in the space-map times of *T*. Near these zones, a kind of backbone pattern and a foliar structure appear in for |*H*| in Fig. 4a,b, respectively. All these structures result from the interaction of Turing and Hopf modes.

The qualitative correspondence between the stability diagram and the numerical solution is also exemplified in Fig. 5a,b, where the stability diagram is qualitatively similar in both cases. However, we have proved numerically that the Eckhaus instability occurs in Fig. 5b for shorter domains as predicted in our formalism. It is also confirmed in the numerical simulations that the solution with *R* = *Q* = 0 is unstable for any domain size in both cases. This statement was also proved for some parameter values of a reaction-diffusion system, where phase instability can occur even for very short domains (see ref.^12^). In these Figures, patterns resemble those founded in refs.^29–31^ for defect chaos and temporal intermittency.

Finally, in Fig. 6 we illustrate the phase chaos. This study suggest the appealing possibility of using the coupled real and complex GL equations in (2) for the study of intermittency and chaos already studied for the sole complex GL equation (see refs^30,31^) for dynamical systems near the CTHP, where both behaviors appear together with a prevailing spatial pattern.

Therefore, by means of numerical simulations, we have identified some qualitative relations between the size of the domain and the stability diagrams. We show that sometimes the homogeneous state is always unstable (Fig. 5), that in some systems the Eckhaus instability cannot occur without a phase unstabilization (Figs 4a and 5), and that the sizes of the domain where any instability occurs are directly related to the position of the unstable regions on the stability diagrams (by comparing Fig. 5a with Figs 5a and 1b with 6). Finally, with the numerical simulations we were able to detect that the coupling between Turing and Hopf modes gives place to structures not displayed by the real or complex GL equation separately, as the foliar and backbone patterns of Fig. 4.

Needless to say that these aspects predicted by the stability diagram should be corroborated numerically by the methodology used for Fig. 2a. However, we expect that the qualitative predictions provided by these examples will help to understand general features of the solutions in dynamical system near the CTHP.

## Discussion

In this work we have provided analytic expressions for the Eckhaus and BFN instabilities in dynamical systems near the CTHP, which allowed to understand the change of wavenumber of the spatial pattern and the unstabilitization of the phase in the Mixed mode solution. A linear stability analysis of the perturbed oscillatory pattern solutions of the Turing-Hopf amplitude equations, yielded a fourth order polynomial dispersion relation (13) for the growth of the sideband perturbations. This polynomial was solved using standard perturbation techniques, thus the conditions for the stability of Mixed mode solutions with wavenumber *R* and *Q* in (3) were stablished. In order to verify the validity of our results, equations (19) and (20) were tested in three different ways. First we have computed (19) and (20) in the limit of no coupling between Turing and Hopf modes, and the well known Eckhaus and BFN criteria of stability were recovered. Second, the predicted stability of the solutions in (3) was compared with the numerical simulations of (2), obtaining pretty good agreement. Finally, we show that the obtained stability diagrams are useful to understand some qualitative features of the numerical solutions as the size of the domain increases.

Our theoretical results are useful to understand diverse spatiotemporal solutions of a system near the CTHP, reported in numerical simulations and experiments. In particular, they corroborate that inhomogeneous perturbations give place to changes in the wavenumber of the spatial pattern and to undulations of the phase recently reported by us in a numerical study of a RD system^12^. Our present work allows us to relate them to generalizations of the Eckhaus and BFN instabilities of the MM solution, valid when the system is near the CTHP. Regarding consistency, we also show how all the known results concerning the existence and stability conditions of the MM solution to homogeneous perturbations^3,5,10^ are recovered as limit cases.

More importantly, our analysis of the MM solution allows to construct the stability diagram of the solutions with wavenumbers *R* and *Q* using, besides the existence conditions implicit in (4), the stability results of the MM solution to inhomogeneous perturbations. Our methodology to quantify the stability of the solutions is a useful tool to identify the relationship between the domain size and both the phase instability and the change of the wavenumber of the spatial pattern, since these occur until a critical domain size, capable to fit wave modes that are destabilized by the Eckhaus and/or BFN instability, is reached. We validate our stability diagrams by means of numerical simulations of (2) for a set of parameters, giving a support to our theoretical findings, but further numerical work in this line is still pending.

In view of this, future investigations must be directed to establish the validity of (19) and (20) for a wider range of parameters, mainly away from the CTHP. Besides, although the scope of our results is limited to one-dimensional systems, we believe that the perturbative technique used in this work can be extended to study another type of systems of GL type.

In this work, we have shown the presence of the secondary instabilities from two kinds of experiments. We have shown that if the amplitude of the random perturbations is small compared with that of the tested modes, for example, the solution with wavenumbers (*R*, *Q*) = (0, 0), the result is a stable and homogeneous solution verified in Fig. 2a. This stability assumes that the amplitudes of *T* and *H* in the initial condition are much greater compared with that of the perturbations. However, for Fig. 1, the amplitudes of the mode with (*R*, *Q*) = (0, 0) is almost the same that for any other combination (*R*, *Q*) chosen randomly. In this case, what the system experiences is a competing of modes^32^ which cannot be analyzed with either (12) or (19) and (20). However, as we have discussed in the previous section, these two formulas for predicting secondary instabilities provide a qualitative picture of what are the possible solutions of systems not prepared with any particular choice of initial conditions as the sizes of the domain increases. Our results show that by increasing the sizes of the domain the apparition of secondary instabilities is facilitated. The form of the solutions seems to be related to the intensity of the random perturbations and the sizes of the domain. How these two factors are related to the multiplication of Fourier modes in the solution and what is their relation with multiple modes of the perturbations remain as unsolved and interesting problems that should be addressed for the full understanding of the dynamical system.

It is very important to realize that our results on the stability of the solutions of the Turing-Hopf amplitude equations can be translated to the study of a particular dynamical system, for example a RD system. In this case, the difficulty lies in to determine how a single initial condition in the RD system gives place to two instabilities represented by the combination of two wavenumbers in (3). This is an unsolved problem. One would expect that the selection of the phase wavenumber, *Q*, and of the spatial modulation of the pattern, *R*, occurs by the proximity of these two wavenumbers to the bifurcation values, *i*.*e*., *k* = 0 for the limit cycle solution and *k* = *k*_*c*_ for the Turing pattern. Therefore perturbations of a initial condition similar to (8): ${\bf{u}}\approx Z{e}^{i({k}_{c}+R)x}{{\bf{u}}}_{T}+A{e}^{iQx}{{\bf{u}}}_{H}$ + c.c., should be studied when *k*_*c*_ is relatively large, as compared with *R* and *Q*, and therefore, each wavenumber can be related to the Turing and Hopf modes, respectively. However, this hypothesis for RD systems have to be tested in future works and constitutes a very interesting research line.

We believe that our work opens the way to future research linking the solutions of a RD system near the CTHP, the solutions of the amplitude equations in (2) and the stability predictions of the MM solution provided here. Finally it should be said that some of our numerical simulations of (2) encouraging to pursue in the study of intermittency and chaos over systems where a spatial pattern prevails, and to study some specific structures not arising in the separate real and complex GL equations.

## Supplementary information

Supplementary information
