# Supplementary material for: Spatio-temporal secondary instabilities near the Turing-Hopf bifurcation
Source: Sci Rep. 2019 Aug 2;9:11287. doi: 10.1038/s41598-019-47584-9 (PMC6677724; doi:10.1038/s41598-019-47584-9)
Supplement: Supplementary file 1 — Supplementary information [file 41598_2019_47584_MOESM1_ESM.pdf]

SUPPLEMENTARY INFORMATION to: Spatio-temporal secondary instabilities near the Turing-Hopf bifurcation

Aldo Ledesma-Durán and José Luis Aragón

Centro de Física Aplicada y Tecnología Avanzada,
Universidad Nacional Autónoma de México.
Boulevard Juriquilla 3001 Juriquilla 76230 Querétaro, Mexico.

Here the details on the steps of the perturbative method are provided, including the full expressions of the coefficients used in the dispersion relation.

In what follows, equations with numbers without the capital letter A corresponds to the equations on the main paper.

I. THE PERTURBATIVE APPROACH

I.1. Coefficients of the dispersion relation

The substitution of (12) in (2) yields the eigenvalue problem: $(\mathbf{M}-\lambda\mathbf{I})\mathbf{x}=0$, where $\mathbf{x}=(p,\bar{q},r,\bar{s})^T$ is the vector of perturbations, $\mathbf{I}$ is the 4×4 identity matrix and $\mathbf{M}$ is the matrix of coefficients:

$$\mathbf{M}=\begin{pmatrix} -2a_hA^2-b_h(k+Q)^2+i\Omega-c_hZ^2+\mu_h & -a_hA^2 & -c_hAZ & -c_hAZ \\ -c_hAZ & -2\bar{a_h}A^2-\bar{b_h}(k-Q)^2-i\Omega-\bar{c_h}Z^2+\mu_h & -\bar{c_h}AZ & -\bar{c_h}AZ \\ -c_tAZ & -c_tAZ & -2a_tZ^2-c_tA^2-b_t(k+R)^2+\mu_t & -a_tZ^2 \\ -c_tAZ & -c_tAZ & -a_tZ^2 & -2a_tZ^2-c_tA^2-b_t(k-R)^2+\mu_t \end{pmatrix}.$$

This leads to the eigenvalue problem (13):

$$\lambda^4+f_3(k)\lambda^3+f_2(k)\lambda^2+f_1(k)\lambda+f_0(k)=0 \qquad \text{(A.1)}$$

The polynomials $f_i(k)$, with $i=0,1,2,3$ are given by

$$f_i(k)=f_{i0}+f_{i1}k+f_{i2}k^2+f_{i3}k^3+\cdots \qquad \text{(A.2)}$$

where the coefficients up to third order in k are

$$\begin{aligned}
&f_{00}=f_{01}=f_{02}=0,\quad f_{10}=0, && \text{(A.3)}\\
&f_{33}=0,\quad f_{41}=f_{42}=f_{43}=0,\\
&f_{03}=8iA^2Q(\alpha-\beta)b_t\alpha_r\beta_r\left(Z^2a_t(\sigma\tau\chi-3)-2A^2\tau\alpha_r+2\mu_t\right),\\
&f_{11}=8iA^2QZ^2(\alpha-\beta)a_t\alpha_r\beta_r(\sigma\tau\chi-1),\\
&f_{13}=8iQb_t\beta_r\left(3\beta Z^2a_t+A^2\alpha_r(-\alpha+2\beta\tau+\beta)-2\beta\mu_t\right),\\
&f_{20}=-4A^2Z^2a_t\alpha_r(\sigma\tau-1), && \text{(A.4)}\\
&f_{21}=4iQ\beta_r\left(2\beta Z^2a_t+A^2(\beta-\alpha)\alpha_r\right),\\
&f_{23}=8i\beta Qb_t\beta_r\\
&f_{30}=2\left(Z^2a_t+A^2\alpha_r\right), && \text{(A.5)}\\
&f_{31}=4i\beta Q\beta_r,\\
&f_{32}=2\left(b_t+\beta_r\right), && \text{(A.6)}
\end{aligned}$$

$$\begin{aligned}
f_{12}=4\Big[&-Z^2a_t\left(A^2b_t\alpha_r(\sigma\tau-3)+\beta_r\left(A^2\alpha_r\left(-\alpha\beta-2\beta^2+\beta\gamma\sigma\tau+\sigma\tau-3\right)+2\left(\beta^2+1\right)\mu_h\right)\right)\\
&+2\left(\beta^2+1\right)\sigma Z^4a_t^2\beta_r+2A^2b_t\alpha_r\left(A^2\tau\alpha_r-\mu_t\right)\Big],\\
f_{22}=\;&2Z^2a_t\left(3b_t+2\left(\beta^2\sigma+\sigma+1\right)\beta_r\right)+4b_t\left(A^2(\tau+1)\alpha_r-\mu_t\right)+2\beta_r\left(A^2\left(\alpha\beta+2\beta^2+3\right)\alpha_r-2\left(\beta^2+1\right)\mu_h\right),
\end{aligned}$$

I.2. Recurrence relations

In order to solve (A.1), we use a perturbative approach. So we assume k as a small parameter and the solution λ is written in power series of k as in (14):

$$\lambda=\lambda_0+\lambda_1k+\lambda_2k^2+\lambda_3k^3+\cdots. \qquad \text{(A.7)}$$

This transforms the problem of solving (A.1) in the problem of solving $P_i(\{\lambda_i\}) = 0$ recurrently $(i = 0, 1, 2, \cdots)$, where

$$P_0(\lambda_0)+P_1(\lambda_0,\lambda_1)k+P_2(\lambda_0,\lambda_1,\lambda_2)k^2+P_3(\lambda_0,\lambda_1,\lambda_2,\lambda_3)k^3+\cdots = 0. \tag{A.8}$$

In the general case, each polynomial P_i would depend on the previous coefficients $\lambda_0, \lambda_1, \ldots, \lambda_i$. However, as we will show, the fact that $f_{10} = 0$ in (A.3), will simplify the problem since (8) becomes

$$P_0(\lambda_0) + \lambda_0 \hat{P}_1(\lambda_1)k + P_2(\lambda_0,\lambda_1)k^2 + P_3(\lambda_0,\lambda_1,\lambda_2)k^3 + \cdots = 0. \tag{A.9}$$

Since the linear term is multiplied by λ_0, this term does not provide new information about λ when we consider the bifurcation solutions with $\lambda_0 = 0$. Therefore, it will be necessary to use a higher term in P_{j+1} to find the solution of λ at order k^j. This will allow us to find the solutions of λ recurrently at the desired order.

Solution at order 0 *in* k.- The polynomial at order zero $P_0(\lambda_0)$ is

$$\lambda_0^4 + f_{30}\lambda_0^3 + f_{20}\lambda_0^2 + f_{10}\lambda_0 + f_{00} = 0. \tag{A.10}$$

Since $f_{10} = f_{00} = 0$ in (A.3), this problem is (see Eq. 15)

$$\lambda_0^2\left(\lambda_0^2 + f_{30}\lambda_0 + f_{20}\right) = 0. \tag{A.11}$$

Therefore $\lambda_0 = 0$ is a double root. The other two roots are

$$\lambda_0^s = \frac{1}{2}\left(-f_{30} \pm \sqrt{f_{30}^2 - 4f_{20}}\right). \tag{A.12}$$

In the last equation we used the superscript s to denote the solutions of the stable eigenvalues.

Therefore, from the four solutions λ of (A.1) at order zero, two solutions are bifurcation values and the other two are stable as long as the solutions of $\left(\lambda_0^2 + f_{30}\lambda_0 + f_{20}\right) = 0$ are negative. This occurs if $f_{20}, f_{30} > 0$. From (A.4) and (A.5) these conditions are (16):

$$4A^2Z^2(1-\tau\sigma)\alpha_r\alpha_t > 0, \quad \text{and} \quad 2\left[\alpha_r A^2 + \alpha_t Z^2\right] > 0.$$

Since these conditions are determined at zero order in k, they also determine the stability to homogeneous perturbations of the MM solution. Together with the existence conditions given in (11), these stability conditions of the Mixed mode (MM) solution to homogeneous perturbations were deduced in Ref. [17] of the main text by a simpler methodology.

In summary, the four solutions at order 0 are

$$\lambda_0 = \begin{cases} 0, & \text{Eckhaus} \\ 0, & \text{BFN} \\ -\alpha_r A^2 - Z^2 a_t \pm W, & \text{stable value} \end{cases} \tag{A.13}$$

where $W \equiv \sqrt{(Z^2a_t - A^2\alpha_r)^2 + 4A^2Z^2\tau\sigma\alpha_r a_t}$. Since we are interested in bifurcation solutions, we will assume that the zero order coefficients for the last two eigenvalues are enough to determine their stability and we will focus on the solutions with $\lambda_0 = 0$.

The polynomial at order 1 in k.- The result of $P_1(\{\lambda_i\}) = 0$ using (A.3) is

$$\lambda_0\left(f_{31}\lambda_0^2 + f_{21}\lambda_0 + 3f_{30}\lambda_1\lambda_0 + 2f_{20}\lambda_1 + f_{11} + 4\lambda_1\lambda_0^2\right) = 0. \tag{A.14}$$

For the two stable eigenvalues, the solution at order 1 in k is

$$\lambda_1^s = -\frac{\lambda_0\left(f_{31}\lambda_0 + f_{21}\right) + f_{11}}{\lambda_0\left(3f_{30} + 4\lambda_0\right) + 2f_{20}}. \tag{A.15}$$

By using the given values of f_{ij}, we get

$$\lambda_1^s = -\frac{2iA^2Q\alpha_r\beta_r\left((\alpha+\beta)\left(A^2\alpha_r \pm W\right) - Z^2a_t(-2\alpha\sigma\tau\chi + \alpha + \beta(2\sigma\tau(\chi-2)+1))\right)}{Z^2a_t\left(2A^2\alpha_r(2\sigma\tau-1) \pm W\right) + Z^4a_t^2 + A^2\alpha_r\left(A^2\alpha_r \pm W\right)} \tag{A.16}$$

Since we are interested in the bifurcation values, these solutions won't be considered hereafter.

However, it should be noticed that (A.14) is automatically accomplished for the two bifurcation eigenvalues and thus does not provide new information. It is then necessary to go at higher orders of the polynomial P_{j+1} to obtain λ_j for $j = 1, 2, \ldots$.

Bifurcation solutions at order 1 in k.- From $P_2(\{\lambda_i\}) = 0$, using (A.3) and $\lambda_0 = 0$, we obtain

$$\lambda_1\left(f_{20}\lambda_1 + f_{11}\right) = 0, \tag{A.17}$$

which gives the two different solutions for λ_1 in (17), the trivial solution $\lambda_1^{Eck} = 0$, and $\lambda_1^{BFN} = -f_{11}/(2f_{20})$, which, by the way, is consistent with (A.15) when $\lambda_0 = 0$. In terms of the parameters of the original system (2), this second solution of λ_1 is

$$\lambda_1^{BFN} = \frac{2iQ\beta_r(\alpha-\beta)(1-\tau\sigma\chi)}{1-\tau\sigma}. \tag{A.18}$$

The labels Eck and BFN are included since the respective eigenvalues will be related to Eckhaus and Benjamin-Feir-Newell instabilities, when higher orders of solution in k for λ are included as explained in the text.

Solution at order 2 in k.- From $P_3(\{\lambda_i\}) = 0$, using (A.3) and $\lambda_0 = 0$, we obtain

$$f_{30}\lambda_1^3+f_{21}\lambda_1^2+f_{12}\lambda_1+2f_{20}\lambda_2\lambda_1+f_{11}\lambda_2+f_3 = 0. \quad \text{(A.19)}$$

This equation has a solution for each value of λ_1 given in (17).

For the Eckhaus related eigenvalue, $\lambda_1 = 0$ and therefore $\lambda_2 = -f_{03}/f_{11}$. By replacing the values of f_{ij}, yields

$$\lambda_2^{Eck} = -\frac{b_t\left(Z^2 a_t(\sigma\tau\chi - 3) - 2A^2\tau\alpha_r + 2\mu_t\right)}{Z^2 a_t(\sigma\tau\chi - 1)}. \quad \text{(A.20)}$$

Using Eqs. (8) in terms of Z and R the previous equation can be written as (19):

$$\lambda_2^{Eck} = -b\left[1 - \frac{2b_t R^2}{Z^2(1-\tau\sigma\chi)a_t}\right]. \quad \text{(A.21)}$$

For the second eigenvalue, the solutions for λ_2 using λ_1^{BFN} is $\lambda_2 = \frac{f_3}{f_{11}} + \frac{f_{11}(f_{20}f_{21}-f_{11}f_{30})}{f_{20}^3} - \frac{f_{12}}{f_{20}}$. This yields

$$\begin{aligned}
\lambda_2^{FBN} =& \frac{2\beta_r\left(\alpha^2 - 2\alpha\gamma\sigma\tau + \left(\gamma^2+1\right)\sigma^2\tau^2 - 2\sigma\tau + 1\right)\left(\sigma Z^2 a_t - \mu_h\right)}{A^2\alpha_r(\sigma\tau-1)^3} + \frac{2A^2\sigma\tau(\chi-1)\alpha_r\left(\tau b_t(\sigma\tau-1)^2 + (\alpha-\beta)^2\beta_r(\sigma\tau\chi-1)^2\right)}{Z^2 a_t(\sigma\tau-1)^3(\sigma\tau\chi-1)} \\
& \frac{2\sigma\tau(\chi-1)\left(-b_t(\sigma\tau-1)^2\mu_t - (\alpha-\beta)^2\mu_h\beta_r(\sigma\tau\chi-1)^2\right)}{Z^2 a_t(\sigma\tau-1)^3(\sigma\tau\chi-1)} + \left(\frac{1}{(\sigma\tau-1)^3(\alpha+\sigma\tau(\beta-\gamma)-\beta)}\right)\Big[2\sigma\tau(\alpha-\gamma)b_t(\sigma\tau-1)^2 \\
& + \beta_r(\alpha+\sigma\tau(\beta-\gamma)-\beta)\Big[2\alpha^2\left(\sigma^2\tau+1\right) + \alpha\left(\beta(\sigma\tau-1)(\sigma(2\sigma+1)\tau-1) - 2\gamma\sigma\tau\left(\sigma^2\tau+\sigma+2\right)\right) + 3 \\
& + \sigma\tau\left(-\beta\gamma(\sigma\tau-1)(\sigma(\tau+2)-1) + \sigma\tau\left(2\gamma^2(\sigma+1) - \sigma\tau + 5\right) - 7\right)\Big]\Big]. \quad \text{(A.22)}
\end{aligned}$$

Taking the limit of no interaction with the Turing mode, $\sigma \to 0$, we recover the known BFN criterion in (7). Besides, assuming existence conditions of the MM solution, *i.e.* $A, Z > 0$, we can expand (22) in power series of the coupling between both modes measured by τ. Taking only the leader term we obtain (20).
